# Animal models of disuse-induced bone loss: study protocol for a systematic review

**DOI:** 10.1186/s13643-020-01441-3

**Published:** 2020-08-16

**Authors:** Mikkel Bo Brent, Annemarie Brüel, Jesper Skovhus Thomsen

**Affiliations:** Department of Biomedicine, Aarhus University, Wilhelm Meyers Allé 3, 8000 Aarhus C, Denmark

**Keywords:** Bone loss, Mechanical unloading, Immobilization, Disuse

## Abstract

**Background:**

Disuse is a cardinal sign of various neurological diseases like stroke, cerebral palsy, and amyotrophic lateral sclerosis. Disuse leads to reduced mechanical loading of the skeleton, and a substantial and significant loss of bone mass quickly materializes. Several animal models have been proposed to investigate the pathogenesis of disuse-induced bone loss and to test new pharmaceutical targets to counteract it. As animal models may overcome several of the limitations in observational studies conducted in patients and allow for measurements not possible in humans, the primary objective of the present study is to provide a comprehensive overview of the available animal models of disuse-induced bone loss.

**Methods/design:**

This is a protocol for a systematic review of animal models of disuse-induced bone loss. An exhaustive search will be performed on PubMed and Embase in order to identify relevant studies. The primary outcome will be the method of disuse induction. The secondary outcomes will be related to bone samples and anatomical sites investigated, methods used to analyze and quantify bone loss, and bibliographic information. The protocol adheres to the current guiding principles of the Preferred Reporting Items for Systematic Review and Meta-analysis Protocols (PRISMA-P) 2015 statement. Extracted data will be analyzed with descriptive statistics, and all the methods used to induce disuse will be described in detail with a narrative synthesis.

**Discussion:**

This systematic review will provide an overview of available animal models of disuse-induced bone loss and discuss the different methods used to quantify and analyze the bone loss. Since bone loss caused by disuse is a hallmark of various diseases from different medical specialties, this overview will be of great benefit for all researchers planning to conduct disuse animal studies in the future.

**Systematic review registration:**

PROSPERO CRD42020157452.

## Background

The use of animals to model diseases has a longstanding tradition and is a cornerstone of preclinical research [1]. Animal models provide a unique opportunity to perform experiments under controlled conditions with a uniform population. It enables a framework for high reproducibility, allows to perform measurements not possible in humans, advances science by elucidating disease pathogenesis, and makes it possible to test and explore new pharmaceutical targets [2].

Inability to perform normal weight-bearing and locomotion quickly results in a loss of bone mass due to reduced mechanical stimulation. The rapid nature of bone loss is underlined by several clinical studies reporting dramatic reductions in bone mineral density of up to 8% after few weeks of disuse following severe spinal cord injury [3, 4]. Disuse-induced bone loss is characterized by a reduced bone mineral density, deteriorated trabecular microarchitecture, and subsequent increased risk of complex fractures [5].

Animal models of disuse-induced bone loss have been used since the early twentieth century, where several authors independently have observed bone loss subsequently to nerve transection or limb immobilization with plaster of Paris [6–8]. Since Allison and Brooks conducted the landmark study in 1921 comparing the effect of brachial plexus transection and immobilization using plaster of Paris in dogs [7], several other animal models have been proposed like limb immobilization using elastic bandages [9], the widely used hypokinetic hindlimb unloading model mimicking spaceflight invented by Morey-Holton from the late seventies [10], and the chemical nerve denervation by botulinum toxin injections proposed by Chappard et al. in 2001 [11] to study the effect of disuse and non-weight bearing on bone tissue [7, 10, 11]. Animal models of disuse are important in pre-clinical research to elucidate the underlying pathogenesis of bone loss, discover new targets for pharmaceutical treatment options, and test new treatment regimens. In addition, the use of animal models overcomes some limitations of clinical studies like loss of patients to follow-up, patient invariability, and non-adherence to the study protocol. Another advantage of animal models is the ability to perform more detailed μCT or DXA analyses of bone tissue without radiation concerns and to evaluate whole bone samples instead of small bone biopsies. Since a vast number of human diseases are characterized by reduced mobility and subsequent bone loss [12–14], there is an immense potential for animal models of disuse-induced bone loss in various medical specialties.

The aim of the present review is to give a comprehensive and systematic overview of the various animal models of disuse-induced bone loss, a detailed narrative synthesis of each unique animal model, and a discussion of the methodology used to quantify and analyze the bone loss.

## Methods

This protocol was written in accordance with the current guiding principles outlined in the Preferred Reporting Items for Systematic Review and Meta-analysis Protocols (PRISMA-P) 2015 statement [15] and has been registered in the PROSPERO database (CRD42020157452).

### Research question

This is the first systematic review to provide a comprehensive and complete overview and narrative synthesis of animal models of disuse-induced bone loss and discuss the evolution of methods employed to analyze the bone samples. The following questions will be addressed:

- What kind of animal models are used to study disuse-induced bone loss?
- Which methods are used to analyze bone samples from animals subjected to disuse-induced bone loss?
- Which changes or developments in the methodology of animal models of disuse-induced bone have occurred over time?

### Disease of interest

The diseases of interest are disuse-induced osteoporosis and osteopenia.

### Population studied

This systematic review will examine all in vivo animal models of disuse-induced bone loss.

### Intervention

Disuse-induced bone loss by any form of human intervention that results in altered habitual musculoskeletal function, reduced voluntary movement, or mechanical unloading.

### Outcome measures

The primary outcome will be the method of disuse induction and any related methodological details about it. The secondary outcomes will be bone samples and anatomical sites investigated, methods used to analyze and quantify bone loss, and bibliographic information about the publication.

### Information sources and search strategy

PubMed and Embase will be comprehensively searched with no starting date restriction or language restriction. The search strategy consists of four blocks. The first block includes keywords for bone and skeleton. The second block includes numerous keywords related to disuse. The third block contains the search filter developed by Hooijmans et al. for PubMed [16] and by de Vries et al. for Embase [17] to include all studies on animal experimentation. Finally, the fourth block excludes non-original research publications (Table 1). In addition, Google Scholar and personal file archives will be searched for relevant publications not indexed in PubMed or Embase. The search strategy was developed in close collaboration with an expert librarian and information specialist in systematic reviews. Following advice from the expert librarian, we chose not to include MeSH terms or the keywords osteoporosis and osteopenia to limit the number of unrelated articles.

**Table 1:** Search string for PubMed and Embase

	PubMed	Embase
Block 1	(bone*[tiab] OR skeleton*[tiab])	(bone*:ab,ti OR skeleton*:ab,ti)
AND		
Block 2	(immobilisation*[tiab] OR immobilization*[tiab] OR disuse*[tiab] OR “non ambulating”[tiab] OR restraint*[tiab] OR unloading*[tiab] OR “non weight bearing”[tiab] OR “non load bearing”[tiab] OR “non loadbearing”[tiab] OR paralyzed*[tiab] OR paralysed*[tiab] OR monoplegic*[tiab] OR paraplegic*[tiab] OR hemiplegic*[tiab] OR tetraplegic*[tiab] OR quadroplegic*[tiab])	(immobilisation*:ab,ti OR immobilization*:ab,ti OR disuse*:ab,ti OR “non ambulating”:ab,ti OR restraint*:ab,ti OR unloading*:ab,ti OR “non weight bearing”:ab,ti OR “non load bearing”:ab,ti OR “non loadbearing”:ab,ti OR paralyzed*:ab,ti OR paralysed*:ab,ti OR monoplegic*:ab,ti OR paraplegic*:ab,ti OR hemiplegic*:ab,ti OR tetraplegic*:ab,ti OR quadroplegic*:ab,ti)
AND		
Block 3	Search filter for animal experimentation developed by Hooijmans et al. [16]	Search filter for animal experimentation developed by de Vries et al. [17]
NOT		
Block 4	(“letter”[publication type] OR “comment”[publication type] OR “editorial”[publication type] OR review[publication type])	(“conference abstract”/it OR “conference paper”/it OR “conference review”/it OR “review”/it OR “editorial”/it OR “letter”/it OR “note”/it) AND [embase]/lim NOT [medline]/lim

### Study selection

#### Screening phases

All titles and abstracts will be screened for eligibility by two independent reviewers (MBB and JST) using the online abstract screening tool *abstrackr* [18]. Prior to the abstract screening, the two reviewers will perform a pilot screening of 150 random articles from the dataset retrieved from PubMed and Embase in order to ensure uniform interpretation of the inclusion and exclusion criteria. After the initial title and abstract screening, a full-text screening will be performed of the included studies by the same reviewers. Discrepancies will be resolved by discussion or by a third independent arbitrator (MB). The study selection process will be illustrated using a PRISMA flow diagram.

### Inclusion and exclusion criteria

#### Type of study design

Any original pre-clinical research study conducted in vivo in animals may be eligible for inclusion if an animal model of disuse-induced bone loss has been used and if the effect on calcified bone tissue has been investigated.

#### Type of publications

Publications without new original data like letters to the editor, commentaries, editorials, and reviews will be excluded. Solitary case reports and conference proceedings will also be excluded.

#### Type of animals

All animal models of disuse-induced bone loss are eligible for inclusion, regardless of sex, species, strain, co-morbidity, or genetic modifications.

#### Type of intervention

Animals must have been subjected to disuse-induced bone loss caused by human interventions.

Therefore, studies investigating bone loss in relation to normal physiological states of inactively or dormancy like hibernation only will not be examined in this systematic review. Disuse can be any form of intervention resulting in a state of altered habitual musculoskeletal function, reduced voluntary movement, or mechanical unloading.

#### Type of outcome measures

Studies are included if they report any outcome measure related to the effect of disuse on calcified bone tissue. Since the systematic review focus on animal models of disuse-induced bone loss, studies reporting outcome measures only related to non-calcified bone tissue like bone marrow, cartilage, and blood vessels will be excluded.

### Prioritized list of exclusion criteria

#### Title and abstract screening

1. Not an original study
2. Not an animal study
3. Not related to disuse-induced bone loss
4. Not studying outcomes related to bone tissue

#### Full text screening

1. Not an original study
2. Not a full research article or retrievable
3. Not an animal study
4. Not related to disuse-induced bone loss
5. Not looking at outcomes related to bone tissue

### Data extraction, collection, and management

Data from eligible studies will be extracted by MBB and JST using a predefined extraction scheme under the following headings: bibliographic information, animal characteristics, disuse and study characteristics, bone samples, and methods used to quantify and analyze the disuse-induced bone loss. Data from graphs will be extracted using the program WebPlotDigitizer or similar available software. The data extracted is outlined below in *italic*:

#### Bibliographic information

*PubMed ID*, *Embase ID*, *first author*, and *year of publication* will be extracted in order to identify individual studies. *The number of authors* and *journal names* will also be collected.

#### Animal characteristics

*Type of animal*, *strain*, *sex*, *age and mean bodyweight at study start*, *feeding scheme* (pair-fed or *ad libitum*), *co-morbidities*, and *genetic modifications* will be recorded. If numerical values are presented as an interval, the mean value will be calculated and reported instead.

#### Disuse and study characteristics

*The method used to induce disuse*, *number of disuse methods used*, *anatomical body part affected by disuse*, and *duration of the study*; *inclusion and number of animals in the baseline*, *control*, *and disuse groups*, *methods of randomization/stratification* to each group, and *sample size* and *power calculations* to support the used number of animals per group; and finally, *references to previously published articles using the same method of disuse-induction* and *statement of study approval from a relevant animal ethics committee* will also be extracted.

#### Bone samples

Bones investigated listed by *anatomical name*, *bone sites investigated* (*metaphysis*, *epiphysis*, *diaphysis*, *etc*.), and the *total number of bones and bone sites* investigated.

#### Methods used to quantify and analyze the disuse-induced bone loss

Measurement of *bone length*, *dual-energy X-ray absorptiometry* (*DXA*), *single-photon absorptiometry* (*SPA*), *micro-computed tomography* (*μCT*), *synchrotron radiation μCT*, *mechanical testing*, *finite element analysis*, *peripheral quantitative computed tomography* (*pQCT*), *dynamic and static bone histomorphometry*, *immunohistochemistry*, *bone biomarkers in blood and urine*, *ash weight*, *electron microscopy*, and *gene expression*, this list is not exhaustive, and methods not mentioned will be added to the predefined extraction scheme used to store and managed data during the extraction process.

### Data analysis and synthesis

Data will be analyzed with descriptive statistics using GraphPad Prism 8.1.1 (GraphPad Software, San Diego, CA, USA). Interrater reliability will be evaluated using Cohen’s kappa coefficient or percentage of agreement. In addition, all methods of disuse-induced bone loss will be described in detail using a narrative synthesis to give a comprehensive overview. Moreover, the advantages and disadvantages of the respective animal models will be discussed. The use of different animal models will be shown in a diagram to provide a visual overview of the prevalence of the various animal models. Animal model data will also be tabulated subdivided by the method of disuse induction to clearly display differences in sex, strain, study duration, details, technical aspects of disuse induction, etc. Trends in the most used methods and techniques to evaluate bone loss due to disuse will be illustrated in a diagram to provide a brief overview of the technological improvements from the first primitive materials testing machines and bone ashing to the contemporary advanced 3D tools like high-resolution ex vivo and in vivo μCT.

### Risk of bias

Since this systematic review is explorative in its nature and seeks to provide a comprehensive overview of existing animal models, no thorough risk of bias assessment or strength of the body of evidence using GRADE is planned. However, it will be assessed, whether the animals are allocated to groups at random and whether the groups are similar at baseline.

## Discussion

Animal models in pre-clinical research are widely used to elucidate underlying disease pathogenesis and explore new targets for pharmaceutical treatment [1, 2, 19, 20]. Identification and selection of the optimal animal model for an experiment are arguably one of the most critical steps during initial study planning and preparation. Consulting online databases and previously published articles in the pursuit of a comprehensive overview of known animal models can be very time consuming and exhausting [21]. Systematic reviews of animal models are thus highly relevant to assist researchers in identifying and selecting the most suitable animal model. In osteoporosis research, animal models have been used for more than a century and have helped to elucidate the underlying disease mechanisms and explore new targets for treatment. Since several risk factors have been associated with osteoporosis like age [22], menopausal status [23], hormonal dysregulation [24], malabsorption [25], side effects to medication like prednisolone [26], smoking [27], low BMI [28], immobilization [29], and disuse [30], it is crucial to select the appropriate animal model. Previous studies have revealed that the magnitude of bone loss varies between the different models used [7, 11, 31]. In addition, it has also been established that the bone loss observed by a particular disuse model is strain-specific underlining the importance of the genetic background of the animals used [32, 33].

Taking the complexity of osteoporosis and disuse-induced bone loss into consideration, this systematic review seeks to establish a comprehensive overview of animal models of disuse-induced bone loss and the methods applied to quantify and analyze the subsequent changes in calcified bone tissue.

## Strengths and limitations

The strength of the review is a systematic approach to search for articles in two major databases (PubMed and Embase), where the screening for eligible articles are performed by two independent reviewers without any language or date restrictions. The review will provide an extensive overview of animal models of disuse-induced bone loss and assist researchers in choosing the most suitable animal model. The main limitation of the review is that articles not indexed in PubMed or Embase might not be found and assessed for inclusion. Another possible limitation is potentially relevant articles not using keywords or phrases in their titles, or abstracts will not be retrieved by our search string.

## Data Availability

Not applicable.

## Abbreviations

BMI: Body mass index
DXA: Dual energy X-ray absorptiometry
GRADE: Grading of Recommendations Assessment, Development and Evaluation
pQCT: Peripheral quantitative computed tomography
PRISMA-P: Preferred Reporting Items for Systematic Review and Meta-analysis Protocols
SPA: Single photon absorptiometry
μCT: Micro computed tomography
